# High-throughput detection of ethanol-producing cyanobacteria in a microdroplet platform

**DOI:** 10.1098/rsif.2015.0216

**Published:** 2015-05-06

**Authors:** Sara Abalde-Cela, Anna Gould, Xin Liu, Elena Kazamia, Alison G. Smith, Chris Abell

**Affiliations:** 1 Department of Chemistry, University of Cambridge, Lensfield Road, Cambridge CB2 1EW, UK; 2 Institute of Process Engineering, ETH Zurich, Sonneggstrasse 3, 8092 Zurich, Switzerland; 3 Sphere Fluidics, The Jonas Webb Building, Babraham Research Campus, Babraham, Cambridge CB22 3AT, UK; 4 Department of Plant Sciences, University of Cambridge, Downing Street, Cambridge CB2 3EA, UK

**Keywords:** microdroplets, cyanobacteria, biofuels, fluorescence

## Abstract

Ethanol production by microorganisms is an important renewable energy source. Most processes involve fermentation of sugars from plant feedstock, but there is increasing interest in direct ethanol production by photosynthetic organisms. To facilitate this, a high-throughput screening technique for the detection of ethanol is required. Here, a method for the quantitative detection of ethanol in a microdroplet-based platform is described that can be used for screening cyanobacterial strains to identify those with the highest ethanol productivity levels. The detection of ethanol by enzymatic assay was optimized both in bulk and in microdroplets. In parallel, the encapsulation of engineered ethanol-producing cyanobacteria in microdroplets and their growth dynamics in microdroplet reservoirs were demonstrated. The combination of modular microdroplet operations including droplet generation for cyanobacteria encapsulation, droplet re-injection and pico-injection, and laser-induced fluorescence, were used to create this new platform to screen genetically engineered strains of cyanobacteria with different levels of ethanol production.

## 1. Introduction

The wide variety of metabolites that photosynthetic organisms produce is attracting the attention of researchers in academia and industry, with a particular focus on biofuel production [1]. Nowadays, the most common biofuels are biodiesel obtained from oil crops, and ethanol produced by yeast fermentation of sugars from starchy crops such as maize or sugar cane [2]. In particular, bioethanol is emerging as one of the most promising non-fossil energy resources, due to its ability to be a ‘drop-in’ fuel mixed with gasoline (petrol). However, bioethanol production from sugars obtained from arable crops requires high land areas to meet the energy requirements and so competes with land for food production. As a consequence, the need for alternative bioethanol producers is a critical issue in the biofuel field [3,4]. Microalgae and cyanobacteria are potential candidates to circumvent the limitations of crop-based ethanol production due to their oxygenic photosynthesis, higher reported productivity and non-competition for arable land [5]. Specifically, cyanobacteria genetically modified to produce ethanol at enhanced productivity rates appear prime candidates for a sustainable and economically efficient bioethanol-based energy industry. In this scenario, metabolic engineering offers the route to generate strains of cyanobacteria with high ethanol productivities [2]. Particularly, *Synechocystis* sp. strain PCC 6803 (*Synechocystis*) has been used as a model organism for genetic modification due to its natural transformability and has previously been transformed successfully to produce ethanol [6].

The theoretical energetic potential of bioethanol depends on the robust determination of ethanol produced by cyanobacteria. The accurate quantification of ethanol has been an issue of study since the 1980s [7]. Indeed, many of the techniques have been developed for clinical and forensic analyses of breath, saliva, urine, sera or blood [8]. However, none of these approaches provides an integrated platform for high-throughput ethanol detection and quantification. Microdroplet technology has the potential to meet these challenges [9]. The possibility of automation, use of small volumes, isolated environments avoiding contamination, high reproducibility, high throughput, as well as the possibility of droplet manipulation and analysis of individual droplets, have all attracted interest in the technology [10].

A key advantage of microdroplets is the ability to manipulate them so that each droplet is a unique experiment. Pico-injection of new reagents into droplets based on the control of applied electric fields to the desired droplets is one example of such a manipulation [11,12]. Encapsulation of different types of cells in droplets for culturing or screening purposes has been reported [13–16]. Recently, we reported the encapsulation of different microalgal species and their growth kinetics in a microdroplet reservoir under a range of conditions [17]. Growth of microalgae in microdroplets was shown to be comparable to growth in bulk under the same conditions. These results encouraged us to apply microdroplet technology further to detect ethanol production in genetically engineered cyanobacteria.

In this paper, ethanol is detected in microdroplets by means of an enzymatic assay that converts ethanol into resorufin (RF), a highly fluorescent compound. A combination of microdroplet operations, including droplet generation for cell encapsulation, re-injection and pico-injection, facilitated the indirect detection of ethanol via the fluorescence of RF in microdroplets. This protocol was applied to the analysis of genetically engineered cyanobacteria to distinguish ethanol producers from wild-type strains. The results pave the way for the screening of libraries of genetically modified cyanobacteria to identify cells with higher levels of ethanol production.

## 2. Experimental design

**Figure 1.**
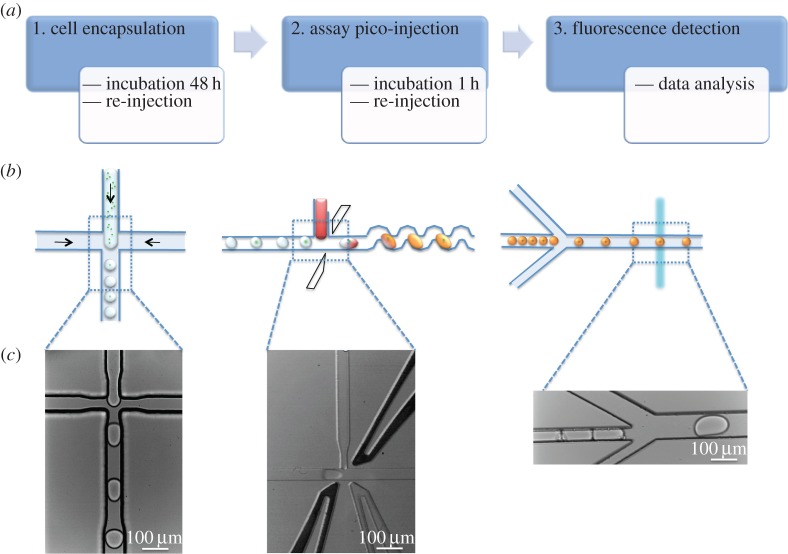
(*a*) Flowchart specifying the steps involved in the study. (*b*) Schematic of each microdroplet operation; from left to right: microdroplet formation for cell encapsulation, pico-injection of assay components in pre-formed droplets, fluorescence detection after ethanol conversion into RF. (*c*) Bright-field images corresponding to each step involved in the process.

Figure 1 describes the three main steps involved in developing a microdroplet-based analytical method to evaluate the ethanol productivity of genetically modified cyanobacteria. The first step comprises the encapsulation of cyanobacteria in microdroplets. Cell encapsulation allows the metabolite of interest, ethanol, which readily diffuses from the cell, to be confined in the microdroplet for assay. The incubation time is optimized to allow the ethanol concentration to accumulate to a level above the sensitivity limits of the optical detection set-up. Incubated droplets are then re-injected in a second microfluidic device, the pico-injection device. This is used to add the reagents to the initial droplet to convert the ethanol into hydrogen peroxide, which reacts with Amplex Red (AR) to form the fluorescent molecule RF. The device incorporates two electrodes to facilitate injection of the reagents into the pre-formed droplets. After an incubation step of 1 h, microdroplets are then re-injected into a third device for fluorescence detection.

## 3. Material and methods

### 3.1. Materials

Unless otherwise stated, all chemicals were purchased from Sigma-Aldrich. Glassware and growth media for cyanobacteria were sterilized by autoclaving. Milli-Q water (Millipore) was used throughout all of the experiments.

### 3.2. Methods

#### 3.2.1. Microfluidic chip fabrication

The microfluidic poly(dimethylsiloxane) (PDMS)/glass device was fabricated by conventional soft lithography methods [18]. Briefly, the device was designed using Autocad 2007 (Autodesk), and a dark-field mask was printed (Microlitho). SU-8 2025 photoresist (Micro-Chem) was spin-coated onto a silicon wafer (diameter: 76.2 mm, Compart Technology Ltd) at 500 r.p.m. for 5 s and then ramped to 1000 r.p.m. at an acceleration of 300 r.p.m. s^−1^ for 33 s. The wafer was subsequently prebaked for 3 min at 65°C and then 9 min at 95°C. It was exposed to UV light through the mask on a mask aligner (MJB4, Suss Microtech) for 10 s. After post-baking for 1 min at 65°C and 4 min at 95°C, the cross-linked features were developed with propylene glycol monomethyl ether acetate, and finally the master was hard-baked for 1 min at 170°C. The final thickness of the photoresist was 75 μm as measured by profilometry. A mixture of PDMS (Sylgard 184) and cross-linker (curing agent, Sylgard 184) (ratio 10 : 1, w/w) was poured over the master, degassed for 30 min and then cured overnight at 75°C. The cured device was cut and peeled from the master, and holes for tubing were made with a biopsy punch (ID = 0.38 mm). After treatment with oxygen plasma for 30 s, the device was bound to another piece of PDMS produced by the same procedure but without holes, in order to close the microfluidic system. The device was baked at 90°C for 1 h to make the sealing permanent. Finally, the microfluidic channels were treated with Aquapel (Pittsburgh, PA, USA), a commercially available fluorosilane, followed by flushing of the channels with fluorous oil.

The fabrication of the pico-injection devices requires introduction of electrodes into predefined channels in the PDMS microfluidic device. This involved heating the device to 140°C on a heating plate. The electrode channels were then filled with 51In/32.5Bi/16.5Sn low-temperature solder (Indium Corporation) melting a small piece of the conductive material inside the appropriate channels [19,20]. Electrical connections with the solder electrodes were made with short pieces of electrical wire. The dimensions and design of the devices used along this work are shown in electronic supplementary material, figure S1.

#### 3.2.2. Cyanobacteria bulk culture

*Synechocystis* sp*.* PCC 6803 wild-type (PCC 6803-wt) strain and the derived strain SAA012, genetically modified to be an ethanol producer, were obtained from Prof. Klaas Hellinwerf, Universiteit van Amsterdam [21]. Each was cultured routinely in BG11 medium [22] in 50 ml conical glass flasks at 30°C under continuous illumination of 40 μmol photons m^−2^ s^−1^ and shaken at 150 r.p.m. NaHCO_3_ (10 mM) was included to act as an inorganic carbon supply for the cells to enable faster growth and be more tolerant to higher light intensities. Phosphate, Na_2_CO_3_ and NaHCO_3_ were autoclaved separately and added after cooling to minimize precipitation. The number of cells in the culture at each stage was determined by using a Bright-Line haemocytometer (Sigma-Aldrich).

#### 3.2.3. Plate reader ethanol assay

To measure ethanol in bulk cultures of *Synechocystis* sp*.*, a custom ethanol assay kit was optimized in a 96-microwell plate by absorbance measurements of the RF band at 570 nm (Power Wave XS, Bio-Tek). The assay kit components were alcohol oxidase (AOX), horseradish peroxidase (HRP) and AR. The microwell plate experiment was designed as shown in electronic supplementary material, table S1. A kinetic procedure was used to collect data every 10 min from 0 to 2 h. Aliquots of each cyanobacteria strain (0.5 ml) were collected at 24 h intervals and the number of cells was determined by cell counting in the haemocytometer. BG11 medium (0.5 ml) was added to replace the extracted volume after aliquot collection. Aliquots were centrifuged and the supernatant was stored in a freezer at −18°C to avoid ethanol evaporation or decomposition.

#### 3.2.4. Resorufin droplet assay

Fluorinated oil HFE-7500 (3M) with Picosurf-1 (PS1, 2.5%, Sphere Fluidics) was used as the dispersed phase, while commercial RF was diluted to various concentrations (0–180 µM) to be the aqueous phase. Liquid flow in all the chips was driven with Harvard Apparatus 2000 syringe infusion pumps. Plastic syringes of 1 and 5 ml were used to load the ethanol solutions and the surfactant-enriched fluorous carrier, respectively. The syringes were connected to the microchips via fine bore polyethylene tubing (ID = 0.38 mm, OD = 1.09 mm, Smiths Medical International Ltd). When flow rates of 2000 µl h^−1^ (continuous phase, fluorinated oil and surfactant) and 250 µl h^−1^ (dispersed phase, being the RF solutions) were used within a flow-focusing nozzle of 80 × 75 (width × height) droplets of ≈100 µm were generated. Droplet formation was monitored through a 10× microscope objective (UPlanFLN, Olympus) and a Phantom V72 fast camera mounted on the microscope (IX71, Olympus). Fluorescence of each droplet was detected as they passed along a laser beam focused on the outlet channel after the droplet generation. A home-made LabVIEW script was used for the quantification of the fluorescence to be adjusted to a calibration curve (electronic supplementary material, figure S2).

#### 3.2.5. Ethanol droplet assay

The pico-injector chip was used to test the developed assay in microdroplets (electronic supplementary material, figure S1B). The same continuous phase as described in the previous section was used at a flow rate of 2000 µl h^−1^. To test the system droplets containing different concentrations (0–160 µM) of ethanol were generated in the pico-injector chip at 200 µl h^−1^. A mixture of the assay kit components with concentrations of AOX and HRP of 0.25 U ml^−1^ and AR of 25 µM was injected through the pico-injection channel at a flow rate of 50 µl h^−1^. Electrodes close to the pico-injection channel were connected to a pulse generator, being parallel-connected, in turn, to an oscillator monitoring the voltage and frequency of droplets and to a high-voltage amplifier. The pulse generator was connected in the ‘run’ mode, so that a continuous voltage was applied to the electrodes via the high-voltage amplifier. A voltage of 200 V at 1 kHz frequency was used for stable pico-injection for 10 min per sample, in order to have enough droplets for a second re-injection. Microdroplets were collected in a 1 ml plastic syringe pre-filled with 0.3 ml of HFE-7500 with 3% PS1. The microdroplets were incubated for 1 h to allow the enzymatic reaction to take place, before re-injection for analysis. The batches of microdroplets were re-injected again into a microchip for laser-induced fluorescence (LIF) detection. The microchip design (specified in electronic supplementary material, figure S1C) comprises an inlet for the re-injected droplets flowing at 100 µl h^−1^ and two inlets for spacing carrier HFE-7500 fluorous oil injected at a flow rate of 500 µl h^−1^.

#### 3.2.6. Cyanobacteria encapsulation

The concentration of the cultures was adjusted to be ≈2.5 × 10^6^ cells ml^−1^ and this was introduced into a microfluidic droplet generation chip as described in §3.2.4 (flow-focusing nozzle of 80 × 75 µm) at a flow rate of 250 µl h^−1^. Fluorinated oil HFE-7500 with PS1 (2.5%) was used as the dispersed phase at a flow rate of 2000 µl h^−1^. The droplets were collected in a 1 ml plastic syringe for further re-injection.

#### 3.2.7. Cyanobacteria on-chip incubation

Microdroplets containing cyanobacteria were generated by the protocol described above. A piece of fine bore polyethylene tubing (5 cm) was used to connect the outlet of the droplet generation chip to a microdroplet reservoir, which was the incubation platform. When the reservoir was filled with microdroplets, it was sealed with two small pieces of closed tubing. The reservoir was placed in a Petri dish containing water to reduce droplet shrinkage, taking advantage of the water permeability of PDMS. The stability of the microdroplets in the reservoir was monitored using a Phantom V72 fast camera every 24 h. Growth kinetics were measured by counting the number of chlorophyll fluorescent cells in droplets at each growth step. The chlorophyll in the cells was detected using an IX71 inverted microscope (Olympus) operated in epifluorescence mode. The fluorescence emission was collected by an objective, filtered (600 nm long-pass edge filter) and finally captured with an EMCCD iXonEM + DU 897 camera (Andor Technology).

#### 3.2.8. Microdroplet re-injection and pico-injection

Microdroplets were incubated in a syringe for 48 h before re-injection. A pico-injector microchip (electronic supplementary material, figure S1B) was used to fuse the assay components into the incubated microdroplets containing cells. A mixture of the assay kit components with concentrations of AOX and HRP of 0.25 U ml^−1^ and AR of 25 µM was injected through the pico-injection channel at a flow rate of 50 µl h^−1^. Pre-formed microdroplets encapsulating cells were re-injected at 200 µl h^−1^ and spaced by HFE-7500 fluorous oil flowing at 2000 µl h^−1^. Electronic connections and protocol for pico-injection were the same as in §3.2.5. A voltage of 200 V at 1 kHz frequency was used for stable pico-injection for 10 min per sample in order to have enough droplets for a second re-injection. Microdroplets were collected again in a 1 ml plastic syringe pre-filled with 0.3 ml of HFE-7500 with 3% PS1. The microdroplets were incubated for 1 h to allow the enzymatic reactions to take place, before re-injection for analysis.

#### 3.2.9. Microdroplet re-injection and detection

The batches of microdroplets were re-injected into a microchip for LIF detection. The microchip design is specified in the electronic supplementary material, and comprises an inlet for the re-injected droplets flowing at 100 µl h^−1^ and two inlets for spacing carrier HFE-7500 fluorous oil injected at a flow rate of 500 µl h^−1^.

#### 3.2.10. Optical set-up and data acquisition

The optical set-up (electronic supplementary material, figure S4, inset) consisted of a laser beam (Picarro Cyan solid state laser; 20 mW, 488 nm) focused through a 40× microscope objective (UPlanFLN mounted). Detection was carried out through the same objective using a photomultiplier tube (H8249, Hamamatsu Photonics). To remove the 488 nm excitation light, the fluorescent light was transmitted through a dichroic beam splitter (FF409-Di02, Semrock) in the microscope filter box. Another dichroic splitter (FF633-Di02, Semrock) placed before the photomultiplier tube was used to split up the orange fluorescence and the white illumination (electronic supplementary material, figure S4), which was used to record pictures and videos using a fast camera (Phantom V72). A band-pass filter (535 ± 120 nm) placed on the photomultiplier was used to remove residual white light going into the detector. Fluorescence was recorded onto a data acquisition card (National Instruments) and analysed using a peak detection algorithm in LabVIEW v. 8.2 (National Instruments).

## 4. Results and discussion

### 4.1. Ethanol assay optimization

**Figure 2.**
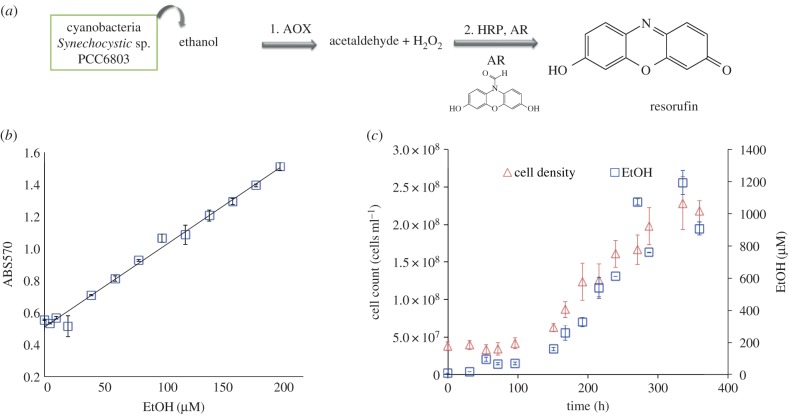
(*a*) Enzymatic reaction assay scheme; AOX: alcohol oxidase, HRP: horseradish peroxidase, AR: Amplex Red. (*b*) Ethanol assay calibration; standard ethanol solution absorbance (squares). (*c*) Ethanol production rate (squares) versus microalgae growth rate (triangles). (Online version in colour.)

For this study, it was necessary to develop a customized ethanol detection assay based on fluorescence. Figure 2*a* shows the chemical steps in the conversion of ethanol into a fluorescent molecule. First, AOX catalyses the conversion of ethanol into acetaldehyde and hydrogen peroxide. The hydrogen peroxidase is then a co-substrate with AR for HRP leading to the formation of fluorescent RF. These coupled enzymatic reactions require an accurate control of the enzymatic conditions to maintain activity [23]. Furthermore, the ratio of reagent concentrations is critical, as low reagent concentrations will limit the conversion of ethanol into RF. On the other hand, too high reagent concentrations in step 2 result in undesired secondary products such as dihydroresorufin and resazurin [23]. Finally, photochemical conversion of AR into RF has been reported under ambient light conditions without the addition of HRP or hydrogen peroxidase [24]. Figure 2*b* shows the calibration of the assay using standard ethanol solutions in a 96-microwell plate. Optimal concentrations of the assay reagents were 0.5 U ml^−1^ for both AOX and HRP and 50 µM AR. These gave a linear absorbance response correlated to ethanol concentration. In parallel, aliquots from an ethanol-producing strain of *Synechocystis* sp. PCC6803 (SAA012) were taken over time to be analysed using the calibrated assay. Samples were frozen at −18°C for storage, to avoid ethanol evaporation, and were kept at this temperature until required. Interpolation of absorbance values obtained for the biological aliquots into the calibration curve is shown in figure 2*b* (triangles). Ethanol concentrations obtained by interpolation were compared to the cell density measured by haemocytometer when aliquots were taken (figure 2*c*).

### 4.2. Ethanol assay in microdroplets

Having established an ethanol assay in bulk, it then needed to be transferred into microdroplets. Electronic supplementary material, figure S2, shows the optical configuration for the detection of RF in a microdroplet platform, as well as the calibration of the system with commercial RF standards. There was a linear relationship between fluorescence intensity and RF over the range of concentrations tested. A trial experiment with microdroplets containing a range of concentrations of ethanol was carried out in order to evaluate the performance of the methodology. Pre-formed microdroplets in a flow-focusing device were re-injected in a second microdroplet device for pico-injection of the ethanol assay reagents. These microdroplets were incubated for 1 h followed by a second re-injection for RF detection. The frequency of droplet analysis was 100 Hz. A total of 2000 droplets were used for the mean and standard deviation calculations. The *Z*′-factor [25] for this assay protocol was 0.86. A linear response of fluorescence intensity was obtained over a range of concentrations (electronic supplementary material, figure S2). Results obtained in this experiment show the potential of the developed methodology to be applied in the detection of bioethanol in microdroplets (electronic supplementary material, figure S2).

### 4.3. Culturing *Synechocystis* in microdroplets: growth kinetics

**Figure 3.**
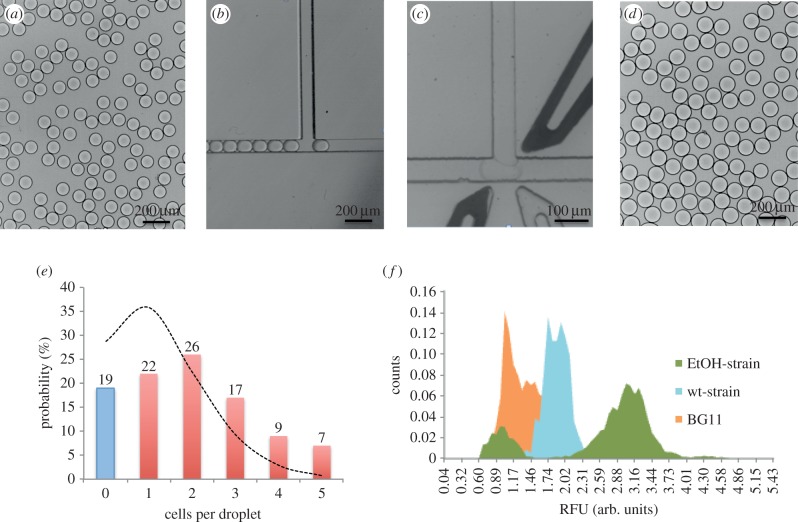
Bright-field images of (*a*) microdroplets after cell encapsulation, diameter = 90 µm. (*b*) Re-injection of microdroplets in a pico-injection device after 24 h incubation. (*c*) A re-injected microdroplet being pico-injected with the assay components for ethanol detection. (*d*) Microdroplets after pico-injection, diameter = 110 µm. (*e*) Histogram showing the number of cells per droplet distribution after encapsulation by using an 80 × 75 µm (w × h) flow-focusing microfluidic chip with flow rates of 2000 and 250 µl h^−1^ for the oil and for the cell suspension, respectively. Discontinuous curve showing the Poisson curve corresponding to a 90 µm droplet (380 pl) for a cell concentration when droplet formation of 2.5 × 10^6^ cells ml^−1^. (*f*) Histogram showing the fluorescence intensity distribution of the microdroplets after detection. In orange, pre-formed droplets just containing the culture media (BG11); in blue, the fluorescence pattern obtained after encapsulating the wild-type strain; and in green, the two-populations profile obtained when encapsulating the ethanol-producing strain.

Cyanobacteria were encapsulated in 90 µm diameter microdroplets and stored in a microdroplet chamber in Picosurfactant-2 (PS2) 2.5% diluted in FC40. Figure 3*e* shows the final distribution of cells in droplets obtained using a 75 × 80 µm (w × h) flow-focusing device and flow rates of 250 µl h^−1^ and 2000 µl h^−1^ for the cell solution and fluorous oil containing surfactant, respectively. The distribution of cells in droplets was defined by Poisson statistics and was analysed by counting the number of cells per droplet in 200 droplets. The discontinuous curve superposed in the histogram of figure 3*e* is the theoretical fitting of the expected Poisson distribution [26]. To the best of our knowledge, this is the first demonstration that *Synechocystis* sp. PCC6803 can be cultured and grown in microdroplets. Dynamic growth of cells in droplets was monitored at 0, 1, 2, 3 and 4 days. Electronic supplementary material, figure S3, depicts the increase in cell density per droplet as the incubation time increases.

### 4.4. Screening ethanol-producing strains in microdroplets

Having established the methodology to detect ethanol in microdroplets and to grow cyanobacteria in microdroplets, it was then possible to use this combined ability to distinguish between different ethanol producers. As proof-of-concept, a genetically modified strain of an ethanol-producing strain of *Synechocystis* (SAA012) [21] was encapsulated in microdroplets using the protocol established in the reservoir test experiments. Two negative controls were used for the validation of the positive results. Microdroplets containing just the growth medium, BG11, were used as the negative control. Also, a batch of microdroplets was generated containing a wild-type *Synechocystis* strain that does not produce ethanol (PCC 6803-wt) and analysed. The flow rate and cell concentration conditions gave 19% of empty droplets and 22, 26, 17, 9 and 7% of microdroplets containing 1, 2, 3, 4 and 5 cells, respectively. The three samples (SAA012, and two negative controls) were incubated for 48 h to allow ethanol accumulation. After this first incubation step, droplets were re-injected for pico-injection of the assay mix and incubated for 1 h to allow ethanol conversion into RF. It is important to note that such combinations of microfluidic operations within droplets need to be done accurately to maintain droplet stability from one step to another, as well as to avoid droplet fusion. Videos of the stability of droplet pico-injection and re-injection steps can be found in the electronic supplementary material. Bright-field images shown in figure 3*a*–*d* show monodisperse droplets after cell encapsulation, the re-injection channel and spacing of droplets to be pico-injected in the same chip, and the final size of droplets after pico-injection (110 µm). The microdroplets were still monodisperse after pico-injection, showing that the pico-injection operation is robust and stable along the process.

Another critical parameter is the applied voltage used for pico-injection. When using high voltages (more than 800 V), droplets coalesced in the outlet channel after pico-injection. On the other hand, when using low voltages (less than 100 V), the pico-injection was not effective. Optimization was carried out to obtain accurate, stable and robust pico-injection; this was achieved at 200 V. Finally, droplets were again re-injected into a microfluidic chip for fluorescence detection of RF. A 488 nm laser was focused in the outlet channel in the detection device and monitored by software supported by LabVIEW. Both negative controls show essentially a single population of cells. Those containing BG11 medium only have low fluorescence, due to the inherent background of the assay components. The PCC 6803-wt cells have higher fluorescence, due to the presence of chlorophyll. By contrast, analysis of 6275 droplets encapsulating the SAA012 is shown in figure 3*f* in green. Two different populations were clearly distinguishable: 22% of the total population of droplets presented low fluorescence, equivalent to that seen for PCC 6803-wt, while 78% of the population were highly fluorescent, corresponding to the presence of ethanol in the microdroplets.

There is a clear correlation between the encapsulation distributions shown in figure 3*e* with the fluorescent distribution obtained after ethanol analysis (figure 3*f*). The accumulation of ethanol inside those droplets containing SAA012 during the incubation period was translated into a higher concentration of RF after the pico-injection of the assay reagents. The empty droplets, containing BG11 only, comprised 22% of the sample and showed background fluorescence.

## 5. Conclusion

We have described the optimization of an ethanol assay in microdroplets, based on the transformation of ethanol into RF. The combination of several microdroplet devices and operations, along with careful droplet manipulation, enabled the quantitative determination of ethanol standards in microdroplets. The electrode-based fusion of the assay components with previously generated ethanol droplets avoids degradation of assay components through photo-bleaching or secondary product formation. This method was applied to the high-throughput analysis of genetically engineered ethanol-producing microalgae, showing the potential of this protocol to be applied in high-throughput analysis and sorting of wider cyanobacteria libraries.

## Supplementary Material

Abalde-Cela et al EtO microdroplets ESM_rsif-2015-0216

## Supplementary Material

Picoinjection

## Supplementary Material

Reinjection

## Supplementary Material

Electronic Supplementary Material (ESM)

## Supplementary Material

Electronic Supplementary Material (ESM)
