# Supplementary material for: High-throughput detection of ethanol-producing cyanobacteria in a microdroplet platform
Source: J R Soc Interface. 2015 May 6;12(106):20150216. doi: 10.1098/rsif.2015.0216 (PMC4424702; doi:10.1098/rsif.2015.0216)
Supplement: Abalde-Cela et al EtO microdroplets ESM_rsif-2015-0216 [file rsif20150216supp1.doc]

**SUPLEMMENTARY INFORMATION:**

**High-throughput detection of ethanol-producing cyanobacteria in a microdroplet platform**

Sara Abalde-Cela,^1^ Anna Gould, ^1, 2^ Xin Liu,^1^ Elena Kazamia, ^3^ Alison G. Smith,^3^ Chris Abell^1^

*^1^Department of Chemistry, University of Cambridge, Lensfield Road, Cambridge, CB2 1EW, UK*

^2^ *Institute of Process Engineering, ETH Zurich, Sonneggstrasse 3, 8092 Zurich, Switzerland*

^3^ *Sphere Fluidics, The Jonas Webb Building, Babraham Research Campus, Babraham, Cambridge, Cambridgeshire CB22 3AT*

^4^ *Department of Plant Sciences, University of Cambridge, Downing Street, Cambridge CB2 3EA, UK*

**Figure ESM-1.** Microfluidic device designs. A) Flow-focusing device for microalgae encapsulation 80x75 μm (T-juction dimensions; w x h) ; B)Pico-injection device 80 x 75 μm (T-juction dimensions; w x h); C) Sorting device used for screening 110x75 μm (T-juction dimensions; w x h).

**Figure ESM-2.** Resorufin and ethanol calibration curves in microdroplets. Inset: Fluorescence optical setup for microdroplet detection.

**Figure ESM-3.** A-E) Bright-field images of cells encapsulated in droplets at growth times 0, 24,48,72 and 96 h; F) Schematic representation of cell encapsulation in droplets and reservoir loading; G) Histogram showing the growth of cells in droplets over time.

**Figure ESM-4.** Graph showing absorbance data after performing the assay to check on ethanol leakage from water to fluorous oil.
