# Supplementary figures and images for: High-throughput detection of ethanol-producing cyanobacteria in a microdroplet platform
Source: J R Soc Interface. 2015 May 6;12(106):20150216. doi: 10.1098/rsif.2015.0216 (PMC4424702; doi:10.1098/rsif.2015.0216)

**A. Droplet generation design**

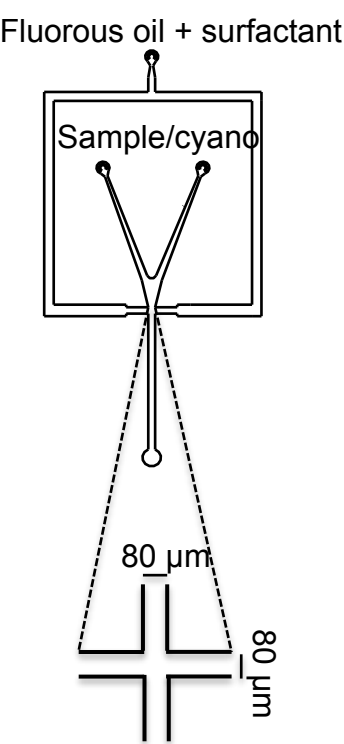

**B. Pico-injection design**

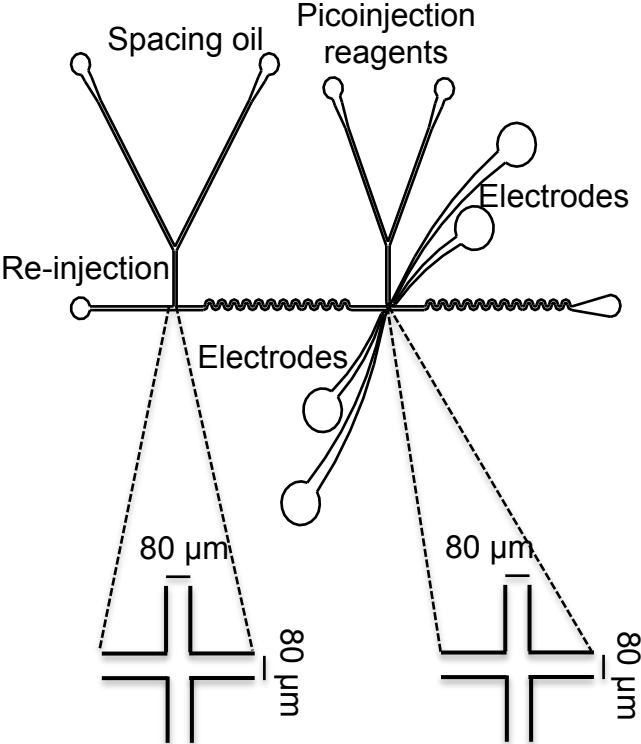

**C. Detection design**

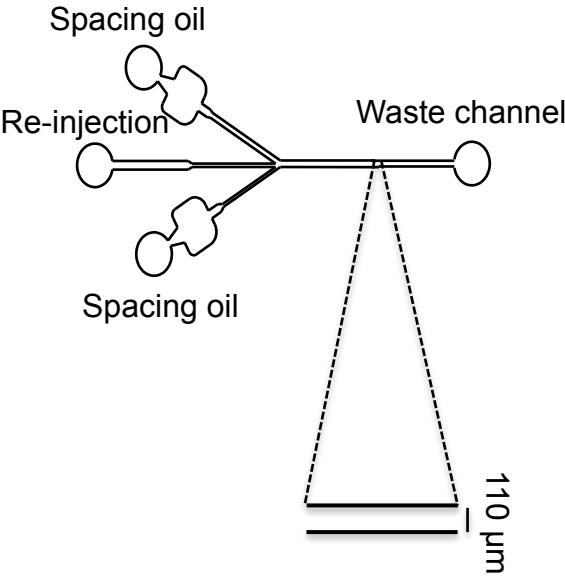

Supplement: Picoinjection [file rsif20150216supp2.pdf]

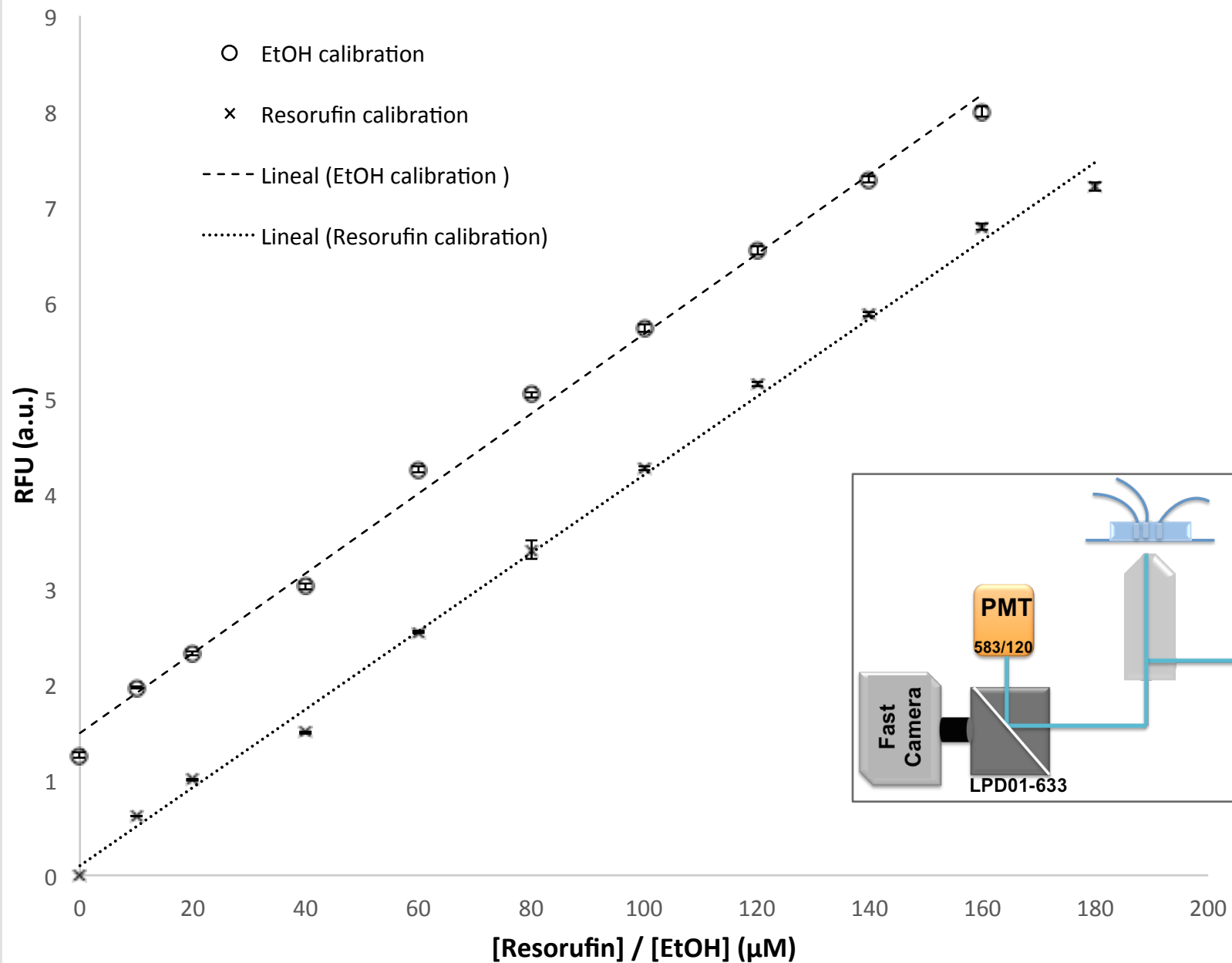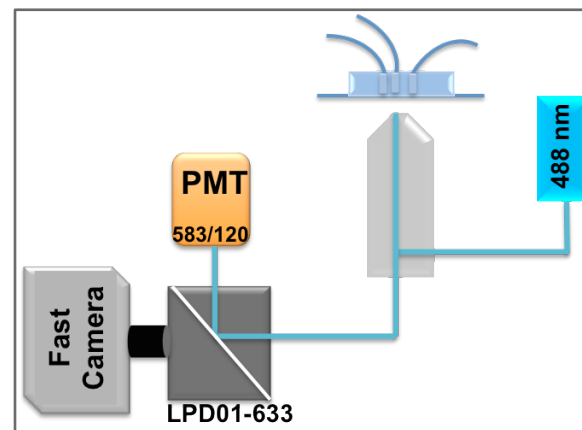

Supplement: Reinjection [file rsif20150216supp3.pdf]

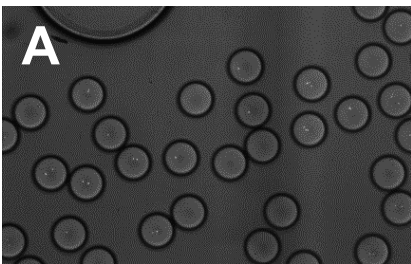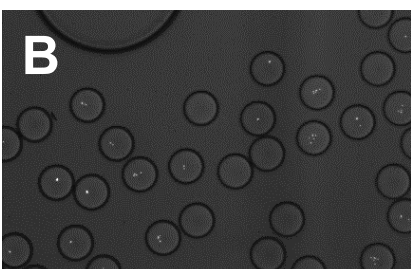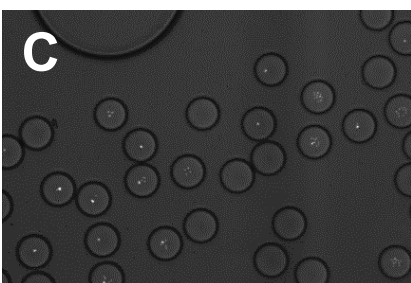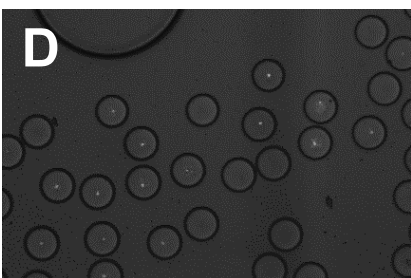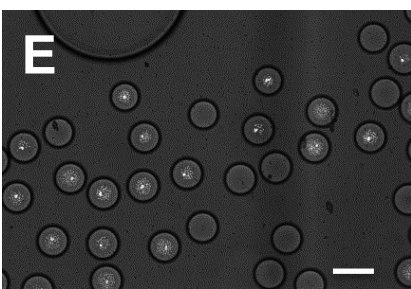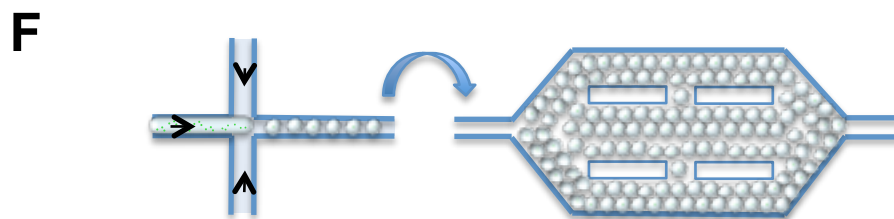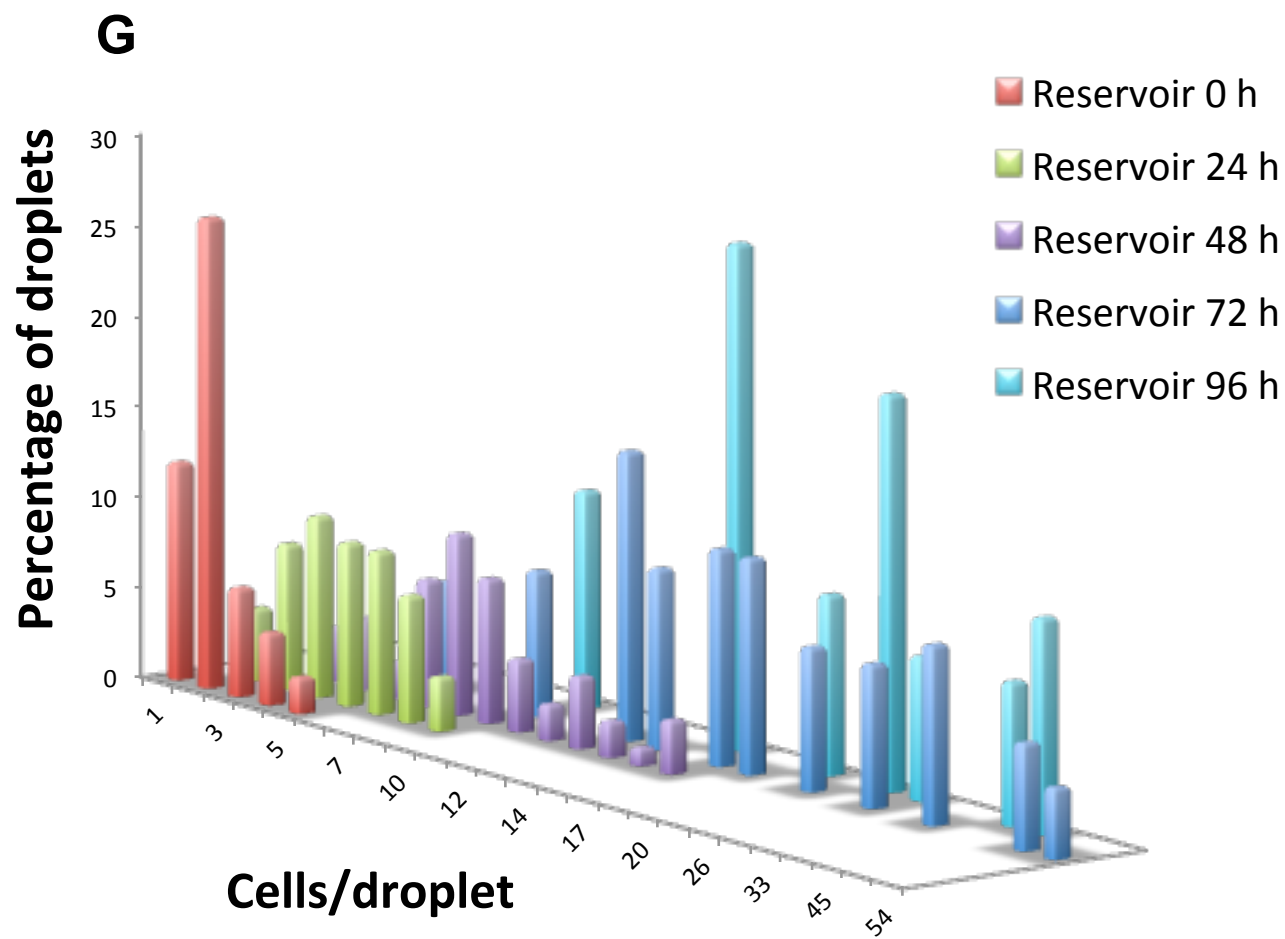

Supplement: Electronic Supplementary Material (ESM) [file rsif20150216supp4.pdf]

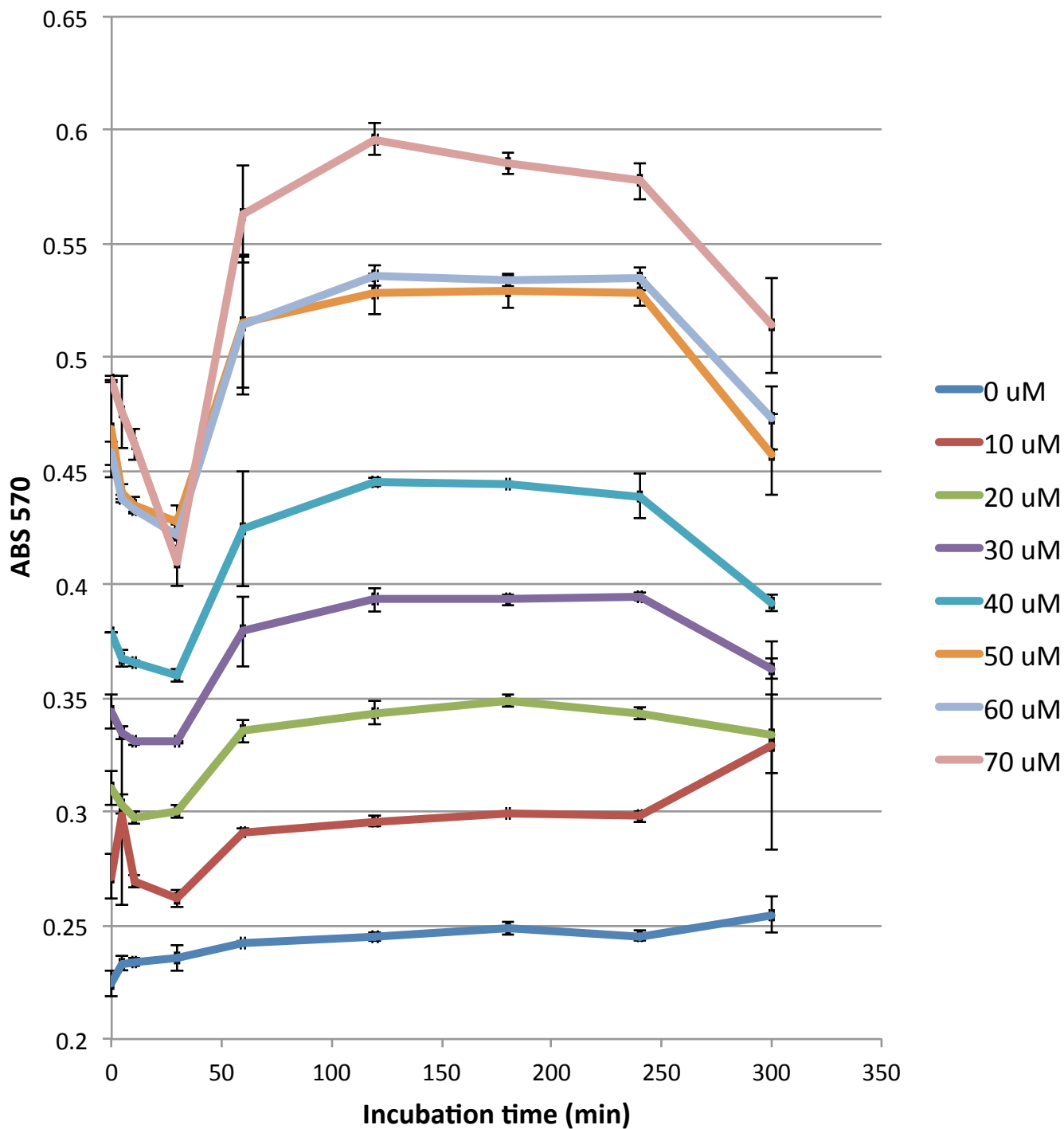

Supplement: Electronic Supplementary Material (ESM) [file rsif20150216supp5.pdf]
